# Novel mutations in TLR genes cause hyporesponsiveness to *Mycobacterium avium *subsp. *paratuberculosis *infection

**DOI:** 10.1186/1471-2156-10-21

**Published:** 2009-05-26

**Authors:** Mangesh R Bhide, Rastislav Mucha, Ivan Mikula, Lucia Kisova, Rostislav Skrabana, Michal Novak, Ivan Mikula

**Affiliations:** 1 Laboratory of Biomedical Microbiology and Immunology, University of Veterinary Medicine, Komenskeho-73, Kosice, Slovakia; 2 Institute of Neuroimmunology, Slovak Academy of Sciences, 842 45 Bratislava, Slovakia

## Abstract

**Background:**

Toll like receptors (TLR) play the central role in the recognition of pathogen associated molecular patterns (PAMPs). Mutations in the TLR1, TLR2 and TLR4 genes may change the ability to recognize PAMPs and cause altered responsiveness to the bacterial pathogens.

**Results:**

The study presents association between TLR gene mutations and increased susceptibility to *Mycobacterium avium *subsp. *paratuberculosis *(MAP) infection. Novel mutations in TLR genes (TLR1- Ser150Gly and Val220Met; TLR2 – Phe670Leu) were statistically correlated with the hindrance in recognition of MAP legends. This correlation was confirmed subsequently by measuring the expression levels of cytokines (IL-4, IL-8, IL-10, IL-12 and IFN-γ) in the mutant and wild type moDCs (mocyte derived dendritic cells) after challenge with MAP cell lysate or LPS. Further *in silico *analysis of the TLR1 and TLR4 ectodomains (ECD) revealed the polymorphic nature of the central ECD and irregularities in the central LRR (leucine rich repeat) motifs.

**Conclusion:**

The most critical positions that may alter the pathogen recognition ability of TLR were: the 9^th ^amino acid position in LRR motif (TLR1–LRR10) and 4^th ^residue downstream to LRR domain (exta-LRR region of TLR4). The study describes novel mutations in the TLRs and presents their association with the MAP infection.

## Background

A conserved set of receptors called pattern-recognition receptors has immense importance in the innate immune system. The role of TLRs, members of mammalian pattern-recognition receptors, has been elaborated in the recent years [1-5]. They are the key components of pathogen recognition mechanism initiating inflammatory responses brought about by microbes or microbial cell components [6,7]. TLR family possesses 14 distinct members identified so far, expressed by epithelial and endothelial cells as well as leukocytes. TLRs are type-I transmembrane receptors composed of an ectodomains, a short transmembrane region, and an intracellular signaling domain that shares homology with that of the IL-1 receptor [8]. TLR mediated cellular activation occurs following the recognition of specific microbial components by the ECD [8]. These receptors act as the sensors for viral, bacterial and fungal structures, for example, TLR3 recognizes viral dsDNA [9], TLR7 and TLR8 recognize the ssRNA [10-12], TLR5 triggers immune signal by detecting flagellin, and CpG DNA is the ligand for TLR9 [13,14]. Toll like receptors focused in this study, TLR1, TLR2 and TLR4, recognize bacterial cell components. TLR2 has shown to mediate the innate immune response to ligands derived from *Mycoplasma*, *Borrelia, Treponema, Chlamydia*, yeasts and parasites [15-20]. TLR2 and TLR4 are critical in the immune response against Gram positive and negative bacteria [21]. Indeed, TLR1 and TLR6 in association with TLR2 (TLR1-TLR2 and TLR2-TLR6 heteromers) recognize a variety of bacterial cell wall components [22-25].

Mutations in the coding region of human TLRs are linked with the altered PAMP recognition ability, signal transduction or innate immune activation in general [19,26-30]. Mutations in TLR1 gene are associated with the variation in the immune response to lipopeptides [27], increased susceptibility to invasive aspergillosis [31] or impaired innate immune sensing of microbial cell wall components [32]. TLR2 and TLR4 gene polymorphisms are often linked with increased risk to infections like tuberculosis [15,33], *Mycobacterium leprae *[34,35], pneumococci or malaria [30,36], urinary tract infections [37] and disease conditions like periodontitis [38], acute rheumatic fever [39] and Crohn's disease [40].

The aim of the study was to screen the ovine population for the mutations in TLR1, TLR2 and TLR4 genes, and to assess their possible association with susceptibility to MAP.

## Results

### Presence of MAP in the sheep population

82 sheep (11.3%) were found to be infected with MAP when tested with ELISA as well as *IS900 *based PCR. MAP infected (n = 82) and non-infected (n = 838) sheep were studied further for the presence of TLR mutations.

### TLRs gene mutations and MAP infection

None of the earlier cited mutations in TLR2 and TLR4 (TLR2 – Pro681His, Arg677Trp, Arg753Gln, and TLR4 – Asp299Gly, Thr399Ile) were found in the sheep population. However, the sequence analysis revealed novel mutations in the ovine TLR2 and TLR4 (Tables 1, 2, 3). We found novel mutation Phe670Leu in TLR2 gene in 56 sheep infected with MAP. 25% of the subjects carrying this mutation in heterozygous state (OR – 4.5) and 7.6% subjects carrying this mutation in homozygous state (OR – 1.1) were MAP infected (Table 2). Another mutation in TLR2 gene at the base pair 2037 (T to C) exchanging leucine against proline (679^th ^amino acid residue) was found in 54 subjects infected with MAP. Both these mutations are located in highly conserved region of TLR2 gene near the known mutation Arg677Trp.

**Table 1:** Missense mutations in the ovine TLR1

		**Wild type frequency**	**Frequency of mutation in heterozygous state**	**Frequency of mutation in homozygous state**
418A>G	Lys140Glu	0.997 (11.4%)	0	0.002 (0%)
431A>T	Asn144Ile	0.997 (11.4%)	0	0.002 (0%)
448A>G	Ser150Gly	0.897 (8.38%)	0	0.10 (43.2%, ***9.08***)^1^
508T>C	Ser170Pro	0.997 (11.4%)	0	0.002 (0%)
517G>R	Glu173 [Lys, Glu]	0.897 (8.38%)	0.10(43.2%, ***9.08***)	0
601A>T	Ile201Phe	0.997 (11.4%)	0	0.002 (0%)
603T>C	Ile201Phe	0.997 (11.4%)	0	0.002 (0%)
658A>G	Val220Met	0.897 (8.38%)	0	0.102 (43.2%, ***9.08***)

^1 ^The first value in the parenthesis indicate the percentage of animals infected with MAP that carries given point mutation, the second value (***bold-italics***) is the Odd's ratio (OR) indicating the possible linkage between the point mutation and increased susceptibility to MAP infection.

**Table 2:** Missense mutations in the ovine TLR2

		**Wild type frequency**	**Frequency of mutation in heterozygous state**	**Frequency of mutation in homozygous state**
1985A>W	Glu662 [Glu, Val]	0.94 (21%)	0.06 (4.7%)	0
2008A>Y	Phe670 [Leu, Phe]	0.55 (6.5%)	0.25 (25%, ***4.5***)	0.20 (7.6%, ***1.1***)^1^
2012A>M	Lys671 [Asn, Thr]	0.99 (11.4%)	0.01 (0%)	0
2013G>T	Lys671 [Asn, Thr]	0.99 (11.4%)	0	0.01 (0%)
2028G>S	Lys676 [Asn, Lys]	0.99 (11.4%)	0.01 (0%)	0
2037T>Y	Leu679Phe	0.57 (6.3%)	0.35 (19.9%, ***2.01***)	0.08 (10.3%, ***1.36***)
2038G>A	Val680Ile	0.998(11.4%)	0	0.002 (0%)
2040C>T	Val680Ile	0.998(11.4%)	0	0.002 (0%)
2090G>R	Arg697 [His, Arg]	0.997 (12.9%)	0.001 (0%)	0.003 (0%)
2111C>y	Ser704 [Ser, Leu]	0.998 (11.4%)	0.002 (0%)	0
2117G>A	Ser706Asn	0.995 (11.4%)	0	0.005 (0%)
2126G>A	Arg709Lys	0.997 (11.4%)	0	0.003 (0%)
2233G>R	Val745 [Ile, Val]	0.995 (11.4%)	0.001 (0%)	0.004 (0%)
2276G>A	Arg759Lys	0.995 (11.4%)	0	0.005 (0%)
2296G>A	Val766Thr	0.998 (11.4%)	0	0.002 (0%)
2297T>C	Val766Thr	0.998 (11.4%)	0	0.002 (0%)

^1 ^The first value in the parenthesis indicate the percentage of animals infected with MAP that carries given point mutation, the second value (***bold-italics***) is the Odd's ratio (OR) indicating the possible linkage between the point mutation and increased susceptibility to MAP infection.

**Table 3:** Missense mutations in the ovine TLR4

		**Wild type frequency**	**Frequency of mutation in heterozygous state**	**Frequency of mutation in homozygous state**
881G>R	Ser294 [Ser, Asn]	0.84 (12.6%)	0.14 (5.7%)	0.02 (0%)
883A>R	Lys295 [Lys, Glu]	0.84 (12.6%)	0.14 (5.7%)	0.02 (0%)
892T>Y	Trp298 [Trp, Arg]	0.84 (12.6%)	0.14 (5.7%)	0.02 (0%)
934G>A	Val312Met	0.998 (11.4%)	0	0.002 (0%)
955T>C	Ser319Pro	0.998 (11.4%)	0	0.002 (0%)
1029T>K	Asp343 [Glu, Asp]	0.993 (11.4%)	0.007 (0%)	0
1032G>S	Lys344 [Asn, Lys]	0.87 (12.6%)	0.12 (3.5%)	0.012 (0%)
1045A>G	Lys349Glu	0.998 (11.4%)	0	0.002 (0%)
1052G>R	Arg351 [His, Arg]	0.87 (12.3%)	0.12 (5.8%)	0.012 (0%)
1066T>Y	Phe356 [Leu, Phe]	0.36 (14.3%)	0.52 (8.2%)	0.12 (16.4%, ***1.64***)^1^
1088A>R	Asp363 [Asp, Gly]	0.84 (12.5%)	0.14 (5.7%)	0.02 (0%)
1091T>Y	Val364 [Val, Ala]	0.84 (12.5%)	0.14 (5.7%)	0.02 (0%)
1097C>S	Thr366 [Thr, Ser]	0.84 (12.5%)	0.14 (5.7%)	0.02 (0%)
1166G>S	Ser389 [Thr, Ser]	0.998 (11.4%)	0.001 (0%)	0
1183G>K	Asp395 [Asp, Tyr]	0.84 (12.5%)	0.14 (6%)	0.002 (0%)

^1 ^The first value in the parenthesis indicate the percentage of animals infected with MAP that carries given point mutation, the second value (***bold-italics***) is the Odd's ratio (OR) indicating the possible linkage between the point mutation and increased susceptibility to MAP infection.

Novel mutation in TLR4 gene associated with the increased susceptibility to MAP infection was located at the base pair T1066C exchanging phenylalanine against leucine (OR – 1.64). Other mutations found in TLR2 and TLR4 genes in this study (Tables 2 and 3) had no association with the increased susceptibility to MAP infection.

Two mutations (Ser150Gly and Val220Met) in TLR1 gene were found in 74 subjects, of that 32 sheep (43.2%) were infected with MAP. Both these mutations occurred simultaneously in all 74 subjects. Apart from these two mutations we found novel mutations in TLR1 at the base pairs: 418 (A to G), 431 (A to T), 508 (T to C), 601 (A to T) and 603 (T to C) (Table 1).

### TLR expression in mutant moDCs

**Figure 1:**
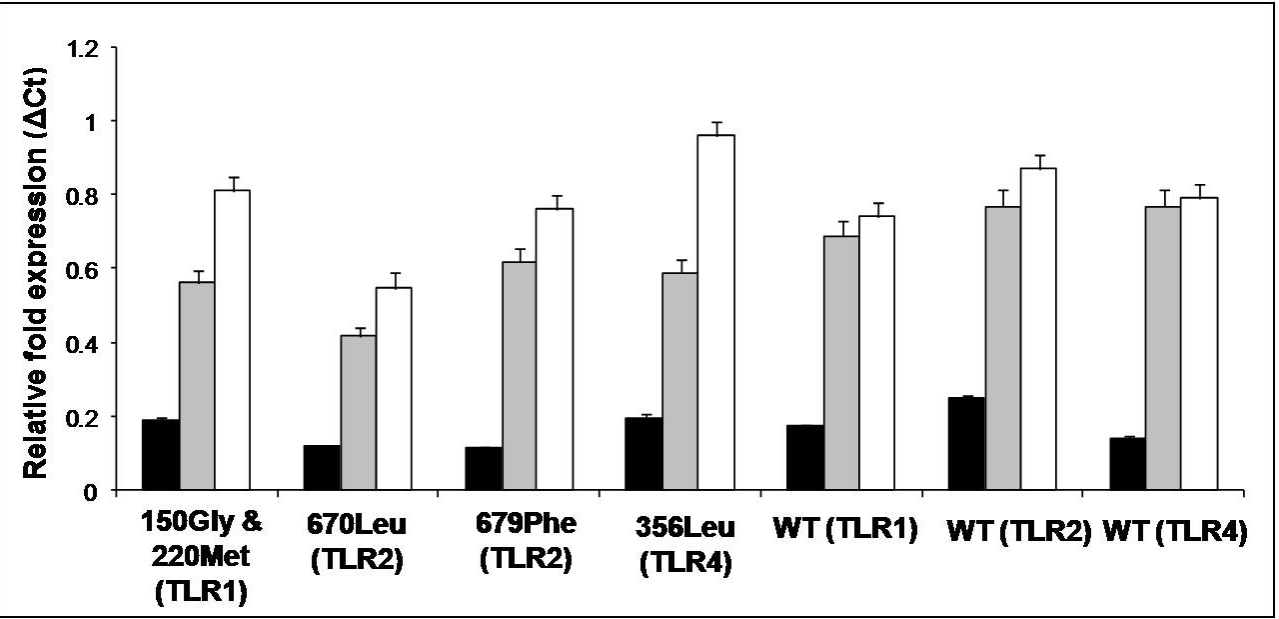
**Activation of TLRs in the mutant and wild type (WT) moDCs challenged with LPS or MAP whole cell lysate**. Relative fold expressions of TLR mRNA in unstimulated (dark bars) and activated mutant as well as wild type moDCs. The cells were either activated by LPS (shaded bars) or MAP whole cell lysate (white bars).

Representative sheep (n = 6 per mutation;*mutant moDCs*), not infected with MAP but carrying mutations associated with MAP infection were included in this phase of the study. Healthy sheep without TLR mutations (n = 6; *wild type moDCs*) were targeted for TLR mRNA expression as a control. We observed 3–6 fold increase in the TLRs expression in activated moDCs as compared to the non-activated control moDCs (Figure 1). However, when mutant and wild type moDCs were challenged with LPS or MAP whole cell lysate, the antigen dependent induction of TLRs was not observed (P > 0.05; Figure 1). Expression of β-actin was unchanged throughout the TLR mRNA expression experiments (data not shown).

### TLR mutations and cytokine mRNA production

**Figure 2:**
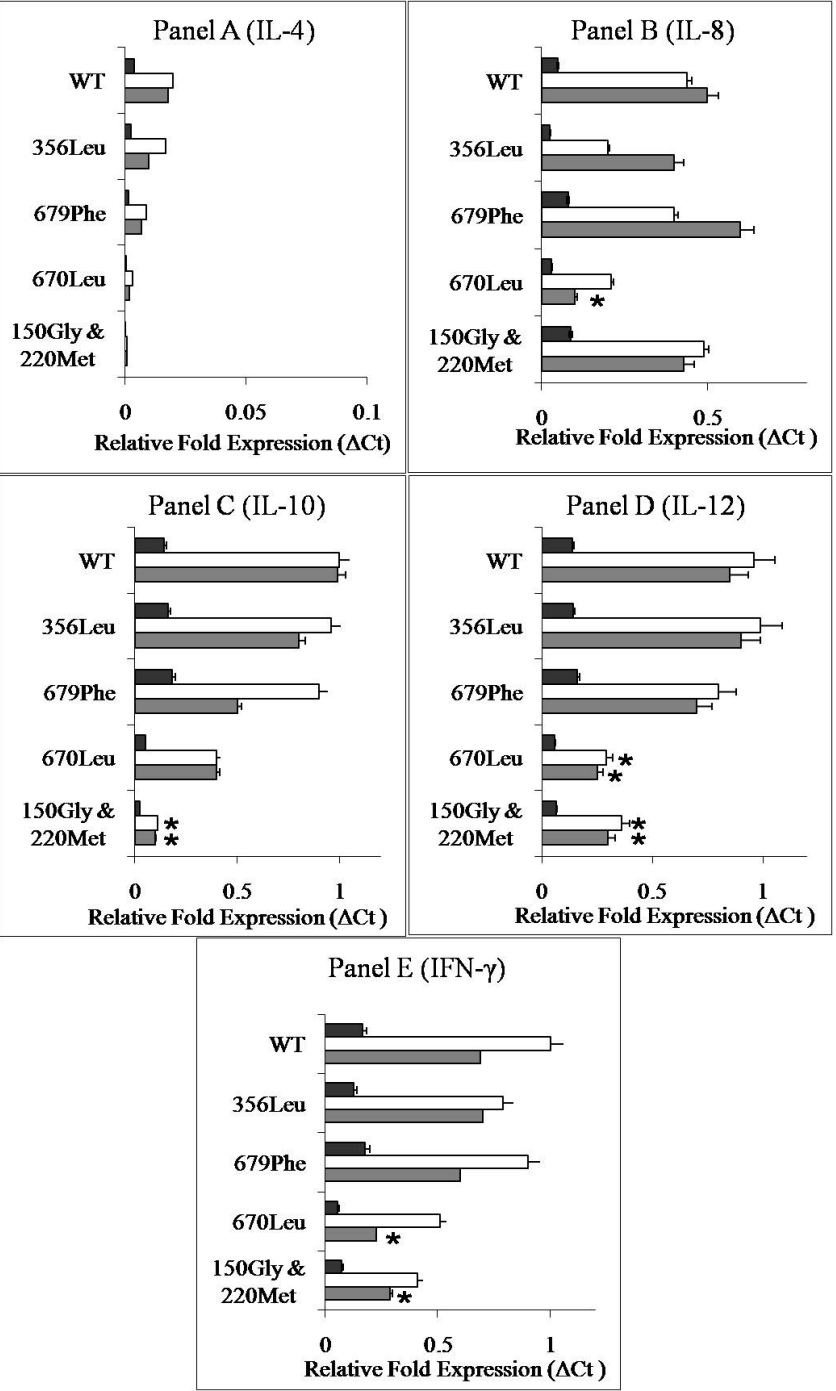
**Expression of cytokine mRNA in the activated mutant and wild type moDCs**. Comparative relative fold expressions of cytokine mRNA in unstimulated (dark horizontal bars) and activated mutant and wild type moDCs. The cells were either activated by LPS (shaded horizontal bars) or MAP whole cell lysate (white horizontal bars). β-actin served as a control gene. Significant difference in the cytokine mRNA expression between stimulated WT and mutant moDCs is depicted by – * (p < 0.05).

IFN-γ, IL-10 and IL-12 were abundantly expressed cytokines in the wild type moDCs when challenged with LPS and MAP whole cell lysate. The cytokines expression, except IL-4, in challenged wild type moDCs was 6 to 9 fold higher than in non-challenged moDCs (Figure 2). In general, MAP cell lysate caused higher cytokine response in the moDCs than LPS. Expression of IL-8 was lower than other abundantly expressed ILs, whereas IL-4 was neither detected in ovine wild type nor in mutant moDCs (Figure 2).

Expression of IL-10 in the challenged TLR1 mutant (Gly150, Met220) moDCs was significantly lower (P < 0.05; Figure 2 panel C) than in the challenged wild type moDCs. This cytokine was also under expressed in the mutant moDCs carrying TLR2 Leu670 mutation (Figure 2 panel C). Another two cytokines, IFN-γ and IL-12, were under expressed in the mutant moDCs carrying TLR1 (Gly150, Met220) and TLR2 (Leu670) mutations compared with the wild type moDCs (Figure 2 panel D and E). Interestingly IL-8 mRNA expression was unchanged in TLR1 mutant moDCs, but significantly lowered (P < 0.05, Figure 2 panel B) in TLR2 Leu670 moDCs. No altered cytokine expression was noticed in the challenged moDCs carrying TLR2 Phe679 and TLR4 Leu356 mutations (Figure 2).

### TLR1 and TLR4 LRR motifs: *In silico *analysis

**Figure 3:**
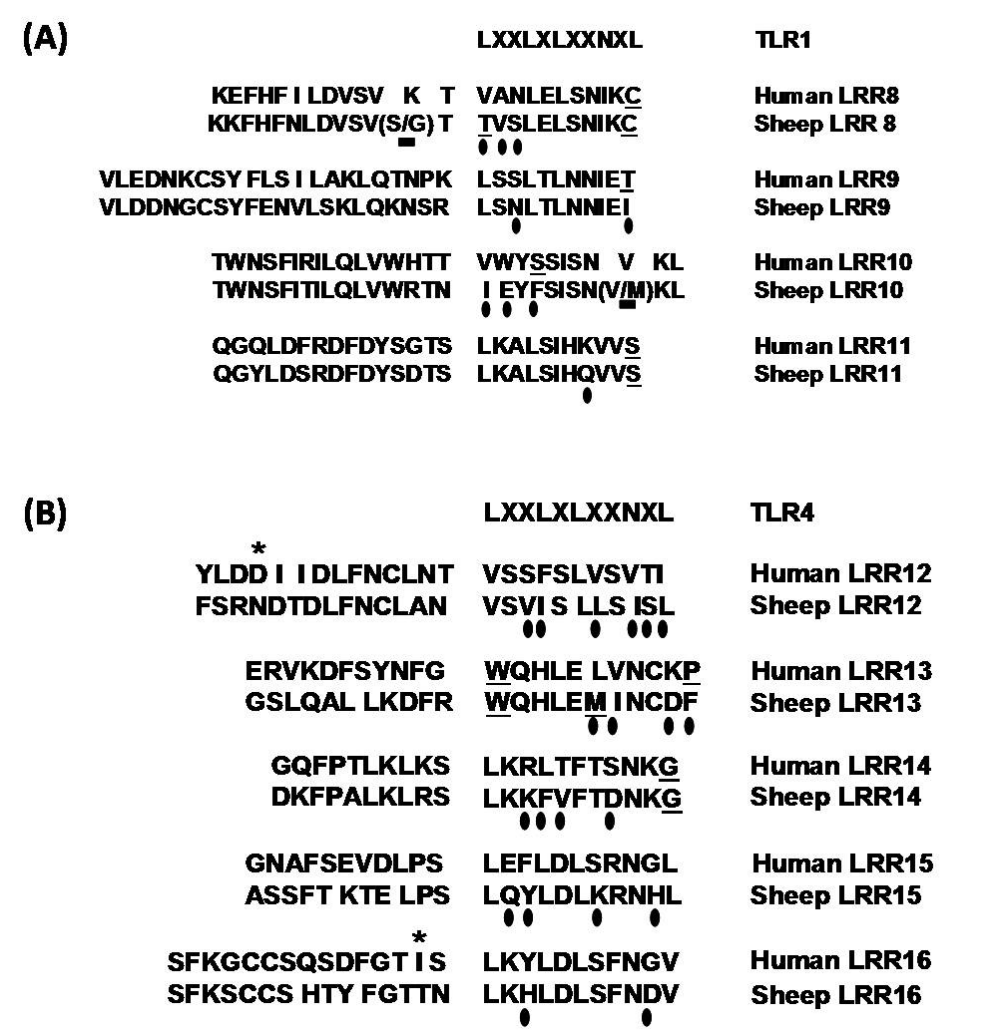
**Comparative amino acid sequences of human and sheep LRR motifs of TLR1 and TLR4**. ***A (TLR1) and B (TLR4)***. Irregularities in the LRR motifs (LxxLxLxxNxL) are depicted with underlined letters; differences between human and ovine LRR domains are indicated with the dark ovals and mutations TLR1- Ser150Gly and Val220Met are highlighted with the dark squares. Known mutations in human TLR4 – Asp299Gly and Thr399Ile are located in extra LRR motif (*, B).

TLR gene family is conservative and show certain homology between human and ovine TLR genes (TLR1 ~75%, TLR2 ~82 and TLR4 ~80). The central core of regular LRR motif is LxxLxLxxNxL, wherein 'x' is any amino acid, 'L' is Leu, Ile, Val or Phe, and 'N' is Asn, Thr, Ser or Cys. Certain irregularities were observed within the LRR motifs of TLR1 (LRR8 and LRR11) and TLR4 (LRR13 and LRR14) (Figure 3 panel A and B). A central part of ovine TLR1 ectodomain (LRR10) was prone to missense mutations and more irregular than other LRRs. TLR1 mutations Gly150 and Met220, causing hyporesponsiveness to MAP infection, were located within the extra-LRR region and intra-LRR motif respectively (Figure 3 panel A). Met220 mutation was found in LRR10 motif at the 9^th ^amino acid position (LxxLxLxx**N**^9th^xL).

We also present LRR motif structure of TLR4 ECD spanning earlier described Asp299Gly and Thr399Ile mutations. Both mutations were found in extra LRR region (Figure 3 panel B) in human TLR4. In the sheep population TLR4 Asp299Gly and Thr399Ile mutations were absent; however, the amino acid position 299 was occupied by asparagine and 399 by theronine.

## Discussion

Recent studies have reported the involvement of TLRs in the innate immune response against MAP [41,42]. Mycobacterial cell wall components like lipomannan, lipoarabinomannan, phosphatidylinositol dimannoside and a 19-kDa lipoprotein are the agonists for the TLR1 and TLR2 receptors [21,43-45], while TLR4 recognizes live *M. tuberculosis *[46]. TLRs mediated downstream pathway leads to the up-regulation of interleukins, chemokines, costimulatory molecules, adhesions and pro-inflammatory/anti-inflammatory cytokines [47,48]. Activation of TLRs not only direct the phagocytic cells to process and present the antigens but also induce their self expression [47]. TLR dependent activation of macrophages leads to the phagocytosis and secretion of inflammatory modulators, whereas activated dendritic cells are directed towards the uptake, its processing and presentation of the antigen to T cells [49].

The heterozygous variant may give ambiguous results. Neither the hyporesponsiveness, nor the reduced cytokine response to TLR agonists was observed in heterozygous TLR4-Asp299Gly [50-52] and heterozygous TLR2-Arg753Gln cells [15,33-35]. Hence, in the present study the *in vitro *challenging was carried out only in moDCs carrying mutations in homozygous state.

In this study, IL-12, IFN-γ and IL-10 were expressed abundantly in the activated wild type moDCs. Others [47,49] have reported that moDCs primarily produce IL-12 upon triggering by the TLR agonists, while macrophages produce IL-10. However, we found no significant difference between the expression levels of IL-12 and IL-10 mRNAs in the activated wild type moDCs. In line with the previous reports [47,53] we also found mixed Th1/Th2 cytokine response in moDCs to TLR agonists. IL-12 and IFN-γ are the Th1 cytokines while IL-10 belongs to the Th2 class. IL-4 was the least expressed Th2 interleukin in the activated wild type as well as in mutant moDCs in this study. Earlier reports [54,55] suggest the structural and functional similarities between IL-13 and IL-4. The lowest expression of IL-4 may be because of its substitution by IL-13. It is important to note that IL-8 mRNA expression was significantly hindered in the activated mutant moDCs possessing TLR2 Leu670 mutation, but not in the activated mutant moDCs carrying TLR1 mutations. This indicates that IL-8 expression might be TLR2 dependent. Supportive data was published earlier, wherein micrococci and peptidoglycan induced transcription of IL-8 in the cells expressing TLR2 only [56].

Human and ovine TLR1 ECD consist of 20 predicted LRRs that take part in the mycobacterial PAMP recognition [57]. The central region of the extracellular domain of human TLR1 (LRR9 to LRR12) is necessary for the sensing of bacterial lipopeptides [32]. We (unpublished data, bovine TLR1 LRR motif analysis) and others (human TLR1) [51] have found that the central part of TLR1 ECD (LRR9 to LRR11) is more irregular and prone to missense mutations. In this study, novel mutation Val220Met was observed in LRR10 motif at the 9^th ^amino acid position (Figure 3A). The presence of methionine in this position may disrupt hydrogen bonds in the LRR loop structure that may cause reduced recognition of PAMPs [51]. The association between mutation at 9^th ^amino acid position in the human TLR-LRR motif and poorly-differentiated gastric adenocarcinomas was reported recently [58]. The increased incidence of MAP infection in sheep bearing Val220Met mutation (43.2%; OR – 9.08) was also observed in this study. Significant reduction (P < 0.05, Figure 2) in the cytokine response to TLR1 agonists (LPS and MAP lysate) in moDCs carrying 220Met (LRR10) and 150Gly (two residues upstream to LRR8) confirms the adverse effect of mutations in the central ECD of TLR1.

In the case of TLR2, the TIR domain is crucial as it forms a TIR-TIR dimerized platform (TLR1-TLR2 and TLR2-TLR6), which promote homotypic protein-protein interactions and further downstream signaling [59]. Hindered expression of the IFN-γ, IL-8 and IL-12 in the moDCs carrying homozygous 670Leu (Figure 2 panel B, D and E) can be due to the impaired dimerization of TLR2-TIR domain with its counterparts. Similar impediment in the IL-12, IL-8 and IFN-γ production was reported earlier in Arg677Trp or Arg753Gln mutants [59-65]. Other crucial residues in human TLR2-TIR domain (713Ser, 730Asp, 748Arg, 749Phe and 752Leu) were reported previously [61].

Mutations in the extra-LRR region may also impede the pattern recognition. 3D structure of the TLR ECD has demonstrated that LRR forms a loop and the juxtaposition of several loops produce solenoid-like structure [51]. The LRR consensus motif forms the inner core of horseshoe structured ECD, while extra LRR regions forms convex surface. Irregularities and/or mutations in the convex surface, for example mutation in 4^th ^residue downstream from LRR motif, may affect PAMP binding onto the TLR horseshoe. The well known human TLR4 mutation, Asp299Gly, is one of the best examples of the mutation at 4^th ^residue downstream from LRR11 (Figure 3B).

## Conclusion

Ser150Gly and Val220Met mutations in TLR1, and Phe670Leu in TLR2 gene were found to cause hindrance in mycobacterial PAMPs recognition. These novel mutations found in TLRs may pose a risk that increases the susceptibility to mycobacterial infection.

## Methods

### Animals

720 pure bred Tsigai sheep, either healthy (healthy cohort) or showing clinical symptoms of paratuberculosis (diseased cohort) were included in this study. The genetic diversity, population structure of this breed is described in details recently [66]. The sheep were from four farms located in the same geographic area (eastern Slovakia). These farms were chosen for the present study because of the high incidence (10–18%) of MAP recorded during paratuberculosis surveillance in the years 2004–2006 (unpublished data). Animals with weight loss and/or chronic diarrhea formed a cohort suspected of paratuberculosis. At least 7 – 8 apparently healthy animals that had close contact with suspected animals were also included in the study. In this way we assured the equal probability of MAP infection on the studied animals. The animal history was recorded and 5–10 ml of the blood (in duplicate) was collected for the serum and buffy coat separation.

### Detection of MAP

Animals were screened for the presence of anti-MAP antibodies in serum as well as for the presence of *IS900 *element of MAP in the buffy coat. Antibodies were detected with Pourquier ELISA paratuberculosis kit (Institute Pourquier, France, ). *IS900 *based nested PCR for MAP detection was designed as described previously [67]. The method, sensitivity and specificity of *IS900 *based PCR are discussed in detail in our previous work [67]. On the basis of PCR and ELISA results, animals were grouped into MAP positive and negative cohorts, and cohorts were subjected to mutation detection in TLR genes.

### Construction of primers and PCR for amplification of TLR gene fragments

ECD of the TLR1 was targeted for mutation detection. Primers were designed (DNASTAR) to amplify a gene fragment covering LRR8 to LRR11 (Table 4). Conditions for PCR were: initial denaturation at 95°C for 3 min, followed by 35 cycles of 94°C for 1.0 min, 52°C for 1 min 20 sec, 72°C for 1.0 min with final extension at 72°C for 10 min. Primers for TLR2 were constructed to amplify gene fragments covering earlier reported Pro681His, Arg677Trp and Arg753Gln mutations [15,39] located in Toll/Interleukin-1 receptor (TIR) domain. Primers designed for TLR4 spanned both previously described major polymorphism sites, Asp299Gly and Thr399Ile [40] located in ECD (LRR11 to LRR16). Nucleotide sequences of TLR2 and TLR4 primers are depicted in Table 4. PCR conditions for TLR2 gene were: initial denaturation at 94°C for 3 min, followed by 35 cycles of 94°C for 60 sec, 56°C for 45 sec, and 72°C for 60 sec with final extension at 72°C for 10 min. The cycling conditions for TLR4 were similar to TLR2 except annealing temperature (57°C).

**Table 4:** Primers used in this study

**Gene**	**Sequence (5'-3')**	**Amplicon length (bp)**	**Annealing temperature (°C)**
*IS900*- MAP (external)	F-AGGGTGTTCGGGGCCGTCGCTTAG R-TGAGGTCGATCGCCCACGTGACCT	406	56.5
*IS900*-MAP (internal)	F-ATGTGGTTGCTGTGTTGGATGG R-CCGCCGCAATCAACTCCAG	298	63.0
TLR1	F-GGAGATACTTATGGGGAAAGAGAA R-GTGTATAGACAAGGCCTTCAGTGA	402	52.0
TLR2	F-CAGGAGCTGGAGCACTTGTACC R-GTCTCATCCACGGGCCAGACCA	362	56.0
TLR4	F-GGGACTGTGCAACCTGACCA R-GCTCTAAGCCCATGAAGTTTGAA	434	53.0
IL-4	F-CCCAGCGCTGGTCTGCTTACT R-GCTTGCCAGGCTGCTGAGATT	283	57.4
IL-8	F-TTGGCCGCTTTCCTGCTCT R-AAATGCCTGCACAACCTTCTGC	249	55.2
IL-10	F-AGCCGAGATGCCAGCACCCTGTC R-AGCTTCTCCCCCAGCGAGTTCACG	293	61.0
IL-12p35	F-GAGCCTGCCCACCACCACA R-GGAAGCCAGGCAACTCTCATT	226	56.4
IFN-γ	F-CTAAGGGTGGGCCTCTTTTCTC R-CATCCACCGGAATTTGAATCAG	237	53.2
β-actin	F-ACTGGGACGACATGGAGAG R-AGGAAGGAAGGCTGGAAGAG	568	54.0

### Single strand conformational polymorphism analysis (SSCP)

Briefly, 5 μl of amplified product was mixed with equal amount of loading dye (98% formamide, 10 mM EDTA, 0.025% bromophenol blue, 0.025% xylene-cyanol), subjected to denaturation at 95°C for 10 min and then cooled rapidly on ice. Denatured single-stranded amplimers were loaded onto 6% acrylamide/bisacrylamide (37.5:1, v/v; Bio-Rad) gels. Electrophoresis was performed using 200 V at 8°C in 0.5% TBE buffer for 20 hours in the electrophoresis chamber (Ingeny, The Netherlands). Gels were silver-stained. Samples were grouped based on SSCP profiles by using Gel-Scan software (BioSciTec, Germany).

### DNA Sequencing

Representative samples from each SSCP genotype were sequenced on an Avant3100 sequencer (Applied Biosystem). The sequences were aligned, then checked for mutations and validated using SeqScape v.2.1 software (Applied Biosystem). Sequences were submitted to the GeneBank (USA) under the accession numbers EF681961 to EF681970. The SNPs were submitted to dbSNP (Genebank) database under the accession numbers: 76880840 to 76880850 and 76878648 to 76878669.

### In-vitro treatment of moDCs with LPS or MAP whole cell lysate

Representative sheep (n = 6 per mutation) which were not infected with MAP but carrying mutations associated with MAP infection in the homozygous state (depicted in tables 1, 2, 3) were included in this phase of the study. Healthy subjects (n = 6) without mutations in TLR were served as controls. moDCs were generated from peripheral blood mononuclear cells as described previously [47] in Nunc 6-well tissue culture plates (approximately 5 × 10^5 ^cells/well in 2 ml of cell suspension). moDCs were either treated with 100 μl of LPS (1 g/ml; Sigma) or 100 μl of MAP whole cell lysate (~890 μg/ml of protein concentration). As a negative control (no cell activation) moDCs were kept untreated. Cells were incubated at 37°C for 4 h in 5% CO_2 _incubator, washed and total RNA was extracted using Purezol RNA isolation kit (Bio-Rad). Complementary DNA (cDNA) was synthesized by using iScript cDNA synthesis kit (Bio-Rad). The cDNA was used for real time PCR to examine the effect of treatment of moDCs on cytokine and TLR mRNA expression. moDCs with or without mutations are designated as mutant moDCs and wildtype moDCs respectively in this report.

### Real time PCR for quantification of TLRs and cytokines mRNA expression

Primers used to amplify cDNAs of the TLRs, cytokines as well as housekeeping gene are depicted in the Table 4. PCR reactions were carried out in triplicate by using iQ SYBR green super mix Kit (Bio-Rad). All PCRs were followed by melting curve analysis (iQ5 thermocycler, Bio-Rad). Melting curve analysis was used to confirm the amplified product purity (confirmation of no non-specific amplicons). For the comparison of gene expression the ΔCt method (relative quantity gene expression) was applied using iQ5 software (Bio-Rad).

### In silico ovine LRR motif analysis

The ovine TLR nucleotide sequences obtained in this study were aligned by ClustalW multiple alignment method (DNASTAR software), translated into putative amino acids and consensus sequences were obtained (BioEdit software). LRR motifs were outlined according to the method described earlier [68] using PFAM and SSpro4.0 servers [69].

### Statistical analysis

The possible linkage between mutation and increased MAP infection was calculated by Odd's ratio (OR) (Win-episcope software). Relative quantity gene expression (ΔCt) was determined as follows -



Where E = efficiency of primer set (% efficiency * 0.01+1), *Ct(control) *= Average Ct for control, and *Ct(sample) *= Average Ct for the sample. Paired t-test (STATGRAPHICS plus 5.1) was used to assess the mutation as well as stimulant (MAP lysate or LPS) dependent variations in cytokine mRNA expression.

### Ethical approval for the use of animals in this study

Although animals were used in this experimental work, no direct experiments were performed on them. Only blood (approximately 20 ml/animal) was collected to detect MAP and mutations. The experiment was neither related with the stem-cell research. Thus no approval from the ethics committee is necessary.

## List of abbreviations

PAMP: pathogen associated molecular patterns; TLR: Toll like receptor; MAP: Mycobacterium avium subsp. Paratuberculosis; ECD: ectodomain; LRR: lucine rich repeat.

## Authors' contributions

MB designed the experiment and carried out real time experiments with LK. The MAP detection was performed by RM. Sequencing and sequence analysis was carried out by IM Jr RS and MN performed *in-silico *analysis. MB with IM Sr prepared this MS. All authors read and approved the final manuscript.

## Authors' informations

The first author and his colleagues are working in the field of Host-pathogen interaction study. Their research includes two members of innate immunity: Toll like receptors and complement regulatory proteins like Factor H. The pathogens which they are dealing with are MAP and *Borrelia *in different hosts system.

## References

[B1] Medzhitov R, Janeway C (2000). Innate immune recognition: mechanisms and pathways. Immunol Rev.

[B2] Akira S (2003). Mammalian Toll-like receptors. Curr Opin Immunol.

[B3] Barton GM, Medzhitov R (2002). Toll-like receptors and their ligands. Curr Top Microbiol Immunol.

[B4] Beutler B (2003). Innate immune responses to microbial poisons: discovery and function of the Toll-like receptors. Annu Rev Pharmacol Toxicol.

[B5] Takeda K, Kaisho T, Akira S (2003). Toll-like receptors. Annu Rev Immunol.

[B6] Li M, Carpio DF, Zheng Y, Bruzzo P, Singh V, Ouaaz F, Medzhitov RM, Beg AA (2001). An essential role of the NF-kappa B/Toll-like receptor pathway in induction of inflammatory and tissue-repair gene expression by necrotic cells. J Immunol.

[B7] Medzhitov R (2001). Toll-like receptors and innate immunity. Nat Rev Immunol.

[B8] Gay NJ, Gangloff M (2007). Structure and function of Toll receptors and their ligands. Annu Rev Biochem.

[B9] Alexopoulou L, Holt AC, Medzhitov R, Flavell RA (2001). Recognition of double-stranded RNA and activation of NF-kappaB by Toll-like receptor 3. Nature.

[B10] Diebold SS, Kaisho T, Hemmi H, Akira S, Reis e Sousa C (2004). Innate antiviral responses by means of TLR7-mediated recognition of single-stranded RNA. Science.

[B11] Heil F, Hemmi H, Hochrein H, Ampenberger F, Kirschning C, Akira S, Lipford G, Wagner H, Bauer S (2004). Species-specific recognition of single-stranded RNA via toll-like receptor 7 and 8. Science.

[B12] Lund JM, Alexopoulou L, Sato A, Karow M, Adams NC, Gale NW, Iwasaki A, Flavell RA (2004). Recognition of single-stranded RNA viruses by Toll-like receptor 7. Proc Natl Acad Sci USA.

[B13] Hayashi F, Smith KD, Ozinsky A, Hawn TR, Yi EC, Goodlett DR, Eng JK, Akira S, Underhill DM, Aderem A (2001). The innate immune response to bacterial flagellin is mediated by Toll-like receptor 5. Nature.

[B14] Hemmi H, Takeuchi O, Kawai T, Kaisho T, Sato S, Sanjo H, Matsumoto M, Hoshino K, Wagner H, Takeda K, Akira S (2000). A Toll-like receptor recognizes bacterial DNA. Nature.

[B15] Ben-Ali M, Barbouche MR, Bousnina S, Chabbou A, Dellagi K (2004). Toll-like receptor 2 Arg677Trp polymorphism is associated with susceptibility to tuberculosis in Tunisian patients. Clin Diagn Lab Immunol.

[B16] Heldwein KA, Liang MD, Andresen TK, Thomas KE, Marty AM, Cuesta N, Vogel SN, Fenton MJ (2003). TLR2 and TLR4 serve distinct roles in the host immune response against Mycobacterium bovis BCG. J Leukoc Biol.

[B17] Janeway CA, Medzhitov R (2002). Innate immune recognition. Annu Rev Immunol.

[B18] Lien E, Sellati TJ, Yoshimura A, Flo TH, Rawadi G, Finberg RW, Carroll JD, Espevik T, Ingalls RR, Radolf JD, Golenbock DT (1999). Toll-like receptor 2 functions as a pattern recognition receptor for diverse bacterial products. J Biol Chem.

[B19] Morre SA, Murillo LS, Bruggeman CA, Pena AS (2003). The role that the functional Asp299Gly polymorphism in the toll-like receptor-4 gene plays in susceptibility to Chlamydia trachomatis-associated tubal infertility. J Infect Dis.

[B20] Underhill DM, Ozinsky A (2002). Toll-like receptors: key mediators of microbe detection. Curr Opin Immunol.

[B21] Underhill DM, Ozinsky A, Smith KD, Aderem A (1999). Toll-like receptor-2 mediates mycobacteria-induced proinflammatory signaling in macrophages. Proc Natl Acad Sci USA.

[B22] Buwitt-Beckmann U, Heine H, Wiesmuller KH, Jung G, Brock R, Akira S, Ulmer AJ (2006). TLR1- and TLR6-independent recognition of bacterial lipopeptides. J Biol Chem.

[B23] Kirschning CJ, Schumann RR (2002). TLR2: cellular sensor for microbial and endogenous molecular patterns. Curr Top Microbiol Immunol.

[B24] Beutler B (2004). Inferences, questions and possibilities in Toll-like receptor signalling. Nature.

[B25] Schumann RR, Tapping RI (2007). Genomic variants of TLR1–it takes (TLR-)two to tango. Eur J Immunol.

[B26] Franchimont D, Vermeire S, El Housni H, Pierik M, Van Steen K, Gustot T, Quertinmont E, Abramowicz M, Van Gossum A, Deviere J, Rutgeerts P (2004). Deficient host-bacteria interactions in inflammatory bowel disease? The toll-like receptor (TLR)-4 Asp299gly polymorphism is associated with Crohn's disease and ulcerative colitis. Gut.

[B27] Hawn TR, Misch EA, Dunstan SJ, Thwaites GE, Lan NT, Quy HT, Chau TT, Rodrigues S, Nachman A, Janer M, Hien TT, Farrar JJ, Aderem A (2007). A common human TLR1 polymorphism regulates the innate immune response to lipopeptides. Eur J Immunol.

[B28] Henckaerts L, Pierik M, Joossens M, Ferrante M, Rutgeerts P, Vermeire S (2007). Mutations in pattern recognition receptor genes modulate seroreactivity to microbial antigens in patients with inflammatory bowel disease. Gut.

[B29] Johnson CM, Lyle EA, Omueti KO, Stepensky VA, Yegin O, Alpsoy E, Hamann L, Schumann RR, Tapping RI (2007). Cutting edge: A common polymorphism impairs cell surface trafficking and functional responses of TLR1 but protects against leprosy. J Immunol.

[B30] Schroder NW, Schumann RR (2005). Single nucleotide polymorphisms of Toll-like receptors and susceptibility to infectious disease. Lancet Infect Dis.

[B31] Kesh S, Mensah NY, Peterlongo P, Jaffe D, Hsu K, M VDB, O'Reilly R, Pamer E, Satagopan J, Papanicolaou GA (2005). TLR1 and TLR6 polymorphisms are associated with susceptibility to invasive aspergillosis after allogeneic stem cell transplantation. Ann N Y Acad Sci.

[B32] Omueti KO, Mazur DJ, Thompson KS, Lyle EA, Tapping RI (2007). The polymorphism P315L of human toll-like receptor 1 impairs innate immune sensing of microbial cell wall components. J Immunol.

[B33] Thuong NT, Hawn TR, Thwaites GE, Chau TT, Lan NT, Quy HT, Hieu NT, Aderem A, Hien TT, Farrar JJ, Dunstan SJ (2007). A polymorphism in human TLR2 is associated with increased susceptibility to tuberculous meningitis. Genes Immun.

[B34] Bochud PY, Hawn TR, Aderem A (2003). Cutting edge: a Toll-like receptor 2 polymorphism that is associated with lepromatous leprosy is unable to mediate mycobacterial signaling. J Immunol.

[B35] Bochud PY, Hawn TR, Siddiqui MR, Saunderson P, Britton S, Abraham I, Argaw AT, Janer M, Zhao LP, Kaplan G, Aderem A (2008). Toll-like receptor 2 (TLR2) polymorphisms are associated with reversal reaction in leprosy. J Infect Dis.

[B36] Khor CC, Chapman SJ, Vannberg FO, Dunne A, Murphy C, Ling EY, Frodsham AJ, Walley AJ, Kyrieleis O, Khan A, Aucan C, Segal S, Moore CE, Knox K, Campbell SJ, Lienhardt C, Scott A, Aaby P, Sow OY, Grignani RT, Sillah J, Sirugo G, Peshu N, Williams TN, Maitland K, Davies RJ, Kwiatkowski DP, Day NP, Yala D, Crook DW, Marsh K, Berkley JA, O'Neill LA, Hill AV (2007). A Mal functional variant is associated with protection against invasive pneumococcal disease, bacteremia, malaria and tuberculosis. Nat Genet.

[B37] Tabel Y, Berdeli A, Mir S (2007). Association of TLR2 gene Arg753Gln polymorphism with urinary tract infection in children. Int J Immunogenet.

[B38] Fukusaki T, Ohara N, Hara Y, Yoshimura A, Yoshiura K (2007). Evidence for association between a Toll-like receptor 4 gene polymorphism and moderate/severe periodontitis in the Japanese population. J Periodontal Res.

[B39] Berdeli A, Celik HA, Ozyurek R, Dogrusoz B, Aydin HH (2005). TLR-2 gene Arg753Gln polymorphism is strongly associated with acute rheumatic fever in children. J Mol Med.

[B40] Hong J, Leung E, Fraser AG, Merriman TR, Vishnu P, Krissansen GW (2007). TLR2, TLR4 and TLR9 polymorphisms and Crohn's disease in a New Zealand Caucasian cohort. J Gastroenterol Hepatol.

[B41] Ferwerda G, Kullberg BJ, de Jong DJ, Girardin SE, Langenberg DM, van Crevel R, Ottenhoff TH, Meer JW Van der, Netea MG (2007). *Mycobacterium paratuberculosis *is recognized by Toll-like receptors and NOD2. J Leukoc Biol.

[B42] Taylor DL, Zhong L, Begg DJ, de Silva K, Whittington RJ (2008). Toll-like receptor genes are differentially expressed at the sites of infection during the progression of Johne's disease in outbred sheep. Vet Immunol Immunopathol.

[B43] Means TK, Lien E, Yoshimura A, Wang S, Golenbock DT, Fenton MJ (1999). The CD14 ligands lipoarabinomannan and lipopolysaccharide differ in their requirement for Toll-like receptors. J Immunol.

[B44] Brightbill HD, Libraty DH, Krutzik SR, Yang RB, Belisle JT, Bleharski JR, Maitland M, Norgard MV, Plevy SE, Smale ST, Brennan PJ, Bloom BR, Godowski PJ, Modlin RL (1999). Host defense mechanisms triggered by microbial lipoproteins through toll-like receptors. Science.

[B45] Krutzik SR, Ochoa MT, Sieling PA, Uematsu S, Ng YW, Legaspi A, Liu PT, Cole ST, Godowski PJ, Maeda Y, Sarno EN, Norgard MV, Brennan PJ, Akira S, Rea TH, Modlin RL (2003). Activation and regulation of Toll-like receptors 2 and 1 in human leprosy. Nat Med.

[B46] Means TK, Wang S, Lien E, Yoshimura A, Golenbock DT, Fenton MJ (1999). Human toll-like receptors mediate cellular activation by *Mycobacterium tuberculosis*. J Immunol.

[B47] Raymond CR, Wilkie BN (2005). Toll-like receptor, MHC II, B7 and cytokine expression by porcine monocytes and monocyte-derived dendritic cells in response to microbial pathogen-associated molecular patterns. Vet Immunol Immunopathol.

[B48] Wills-Karp M (2001). IL-12/IL-13 axis in allergic asthma. J Allergy Clin Immunol.

[B49] Werling D, Hope JC, Howard CJ, Jungi TW (2004). Differential production of cytokines, reactive oxygen and nitrogen by bovine macrophages and dendritic cells stimulated with Toll-like receptor agonists. Immunology.

[B50] von Aulock S, Schroder NW, Traub S, Gueinzius K, Lorenz E, Hartung T, Schumann RR, Hermann C (2004). Heterozygous toll-like receptor 2 polymorphism does not affect lipoteichoic acid-induced chemokine and inflammatory responses. Infect Immun.

[B51] Bell JK, Mullen GE, Leifer CA, Mazzoni A, Davies DR, Segal DM (2003). Leucine-rich repeats and pathogen recognition in Toll-like receptors. Trends Immunol.

[B52] Hamann L, Kumpf O, Muller M, Visintin A, Eckert J, Schlag PM, Schumann RR (2004). A coding mutation within the first exon of the human MD-2 gene results in decreased lipopolysaccharide-induced signaling. Genes Immun.

[B53] Re F, Strominger JL (2001). Toll-like receptor 2 (TLR2) and TLR4 differentially activate human dendritic cells. J Biol Chem.

[B54] de Waal Malefyt R, Figdor CG, Huijbens R, Mohan-Peterson S, Bennett B, Culpepper J, Dang W, Zurawski G, de Vries JE (1993). Effects of IL-13 on phenotype, cytokine production, and cytotoxic function of human monocytes. Comparison with IL-4 and modulation by IFN-gamma or IL-10. J Immunol.

[B55] de Waal Malefyt R, Figdor CG, de Vries JE (1993). Effects of interleukin 4 on monocyte functions: comparison to interleukin 13. Res Immunol.

[B56] Dabbagh K, Dahl ME, Stepick-Biek P, Lewis DB (2002). Toll-like receptor 4 is required for optimal development of Th2 immune responses: role of dendritic cells. J Immunol.

[B57] Ohara T, Morishita T, Suzuki H, Hibi T (2006). Heterozygous Thr 135 Ala polymorphism at leucine-rich repeat (LRR) in genomic DNA of toll-like receptor 4 in patients with poorly-differentiated gastric adenocarcinomas. Int J Mol Med.

[B58] Xu Y, Tao X, Shen B, Horng T, Medzhitov R, Manley JL, Tong L (2000). Structural basis for signal transduction by the Toll/interleukin-1 receptor domains. Nature.

[B59] Gautam JK, Ashish, Comeau LD, Krueger JK, Smith MF (2006). Structural and functional evidence for the role of the TLR2 DD loop in TLR1/TLR2 heterodimerization and signaling. J Biol Chem.

[B60] Kang TJ, Lee SB, Chae GT (2002). A polymorphism in the toll-like receptor 2 is associated with IL-12 production from monocyte in lepromatous leprosy. Cytokine.

[B61] Kang TJ, Yeum CE, Kim BC, You EY, Chae GT (2004). Differential production of interleukin-10 and interleukin-12 in mononuclear cells from leprosy patients with a Toll-like receptor 2 mutation. Immunology.

[B62] Schroder NW, Diterich I, Zinke A, Eckert J, Draing C, von Baehr V, Hassler D, Priem S, Hahn K, Michelsen KS, Hartung T, Burmester GR, Gobel UB, Hermann C, Schumann RR (2005). Heterozygous Arg753Gln polymorphism of human TLR-2 impairs immune activation by *Borrelia burgdorferi *and protects from late stage Lyme disease. J Immunol.

[B63] Mrabet-Dahbi S, Dalpke AH, Niebuhr M, Frey M, Draing C, Brand S, Heeg K, Werfel T, Renz H (2008). The Toll-like receptor 2 R753Q mutation modifies cytokine production and Toll-like receptor expression in atopic dermatitis. J Allergy Clin Immunol.

[B64] Woehrle T, Du W, Goetz A, Hsu HY, Joos TO, Weiss M, Bauer U, Brueckner UB, Marion Schneider E (2008). Pathogen specific cytokine release reveals an effect of TLR2 Arg753Gln during Candida sepsis in humans. Cytokine.

[B65] Tao X, Xu Y, Zheng Y, Beg AA, Tong L (2002). An extensively associated dimer in the structure of the C713S mutant of the TIR domain of human TLR2. Biochem Biophys Res Commun.

[B66] Kusza S, Nagy I, Sasvari Z, Stagel A, Nemeth T, Molnar A, Kume K, Bosze Z, Javor A, Kukovics S (2008). Genetic diversity and population structure of Tsigai and Zackel type of shhep breeds in the Central-, Eastern – and Southern – European regions. Small Ruminant reserach.

[B67] Bhide M, Chakurkar E, Tkacikova L, Barbuddhe S, Novak M, Mikula I (2006). *IS900*-PCR-based detection and characterization of *Mycobacterium avium subsp. paratuberculosis *from buffy coat of cattle and sheep. Vet Microbiol.

[B68] Matsushima N, Tanaka T, Enkhbayar P, Mikami T, Taga M, Yamada K, Kuroki Y (2007). Comparative sequence analysis of leucine-rich repeats (LRRs) within vertebrate toll-like receptors. BMC Genomics.

[B69] Cheng J, Randall AZ, Sweredoski MJ, Baldi P (2005). SCRATCH: a protein structure and structural feature prediction server. Nucleic Acids Res.

